# Antioxidant and anticholinesterase properties of *Echinometra mathaei* and *Ophiocoma erinaceus* venoms from the Persian Gulf

**DOI:** 10.3389/fchem.2023.1332921

**Published:** 2024-01-03

**Authors:** Hamideh Dehghani, Marzieh Rashedinia, Gholamhossein Mohebbi, Amir Vazirizadeh, Neda Baghban

**Affiliations:** ^1^ Department of Pharmacology and Toxicology, School of Pharmacy, Shiraz University of Medical Sciences, Shiraz, Iran; ^2^ The Persian Gulf Marine Biotechnology Research Center, The Persian Gulf Biomedical Research Institute, Bushehr University of Medical Sciences, Bushehr, Iran; ^3^ Department of Marine Biotechnology, The Persian Gulf Research and Studies Center, The Persian Gulf University, Bushehr, Iran

**Keywords:** echinoderm, sea urchin, brittle star, venom, anticholinesterases, antioxidant, secondary metabolite

## Abstract

**Introduction:** The Persian Gulf is home to a diverse range of marine life, including various species of fish, crustaceans, mollusks, and echinoderms. This study investigates the potential therapeutic properties of venoms from echinoderms in the Persian Gulf, specifically their ability to inhibit cholinesterases (Acetylcholinesterase and butyrylcholinesterase) and act as antioxidants.

**Methods:** Four venoms from two echinoderm species, including the spine, gonad, and coelomic fluids of sea urchins, as well as brittle star venoms, were analyzed using various methods, including LD_50_ determination, protein analysis, antioxidant assays, GC-MS for secondary metabolite identification, and molecular docking simulations.

**Results and discussion:** The study’s results revealed the LD_50_ of the samples as follows: 2.231 ± 0.09, 1.03 ± 0.05, 1.12 ± 0.13, and 6.04 ± 0.13 mg/mL, respectively. Additionally, the protein levels were 44.037 ± 0.002, 74.223 ± 0.025, 469.97 ± 0.02, and 104.407 ± 0.025 μg/mL, respectively. SDS-PAGE and total protein studies indicated that at least part of the venom was proteinaceous. Furthermore, the study found that the brittle star samples exhibited significantly higher antioxidant activity compared to other samples, including the standard ascorbic acid, at all tested concentrations. GC-MS analysis identified 12, 23, 21, and 25 compounds in the samples, respectively. These compounds had distinct chemical and bioactive structures, including alkaloids, terpenes, and steroids.

**Conclusion:** These venoms displayed strong cholinesterase inhibitory and antioxidant activities, likely attributed to their protein content and the presence of alkaloids, terpenes, and steroids. Notably, the alkaloid compound **C**_**7**_ was identified as a promising candidate for further research in Alzheimer’s disease therapy. In conclusion, echinoderms in the Persian Gulf may hold significant potential for discovering novel therapeutic agents.

## Graphical Abstract

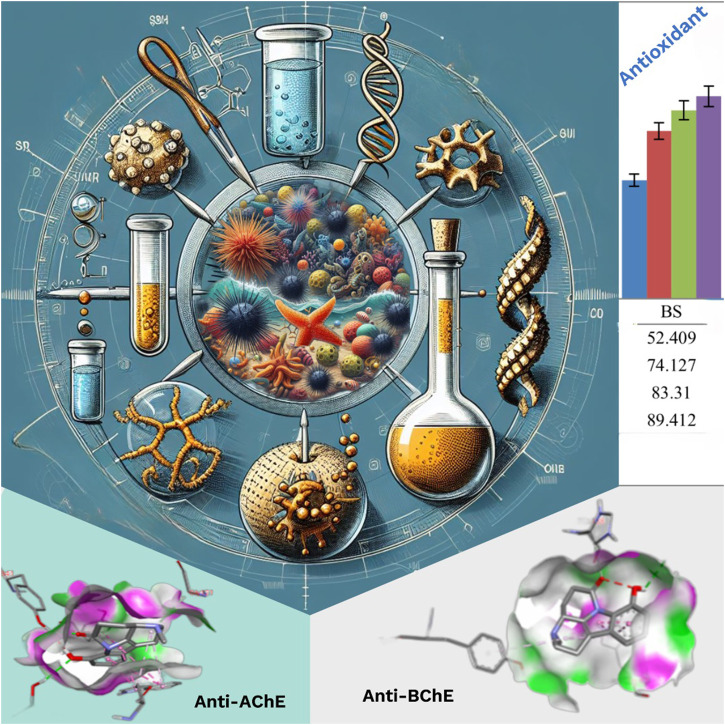

## 1 Introduction

Mother Nature is the first pharmacy to design and synthesize unique drug molecules (Mohebbi et al., 2014). Marine organisms have long been studied for their diverse secondary metabolites with different biological activities, offering superior novelty and bioactivity compared to terrestrial resources (Bayat et al., 2021; Baghban et al., 2022). These secondary metabolites play an important role in the chemical defense of sedentary and slow-moving marine organisms, such as the majority of marine invertebrates (Ebrahimi et al., 2015). Among marine invertebrates, echinoderms, such as sea urchins and brittle stars, stand out for their rich bioactive compounds, including toxins. These compounds play an important role in the chemical defense of these organisms. Some of the echinoderms produce potent toxins that can cause significant lethality in animals (Kalinin, 2021). In recent years, the field of toxinology has witnessed a surge of interest in marine toxins as sources of novel bioactive compounds. These compounds have shown significant potential in drug discovery and development, particularly in the areas of neuropharmacology, oncology, and immunology. Moreover, marine toxins have exhibited remarkable properties such as antioxidant and anticholinesterase activities, which are of considerable importance in the context of human health and disease such as different type of cancers and Alzheimer’s disease (AD) (Abbasi et al., 2012; Chigurupati et al., 2018; Mohebbi et al., 2018; Darabi et al., 2020; Kotzaeroglou and Tsamesidis, 2022).

The Persian Gulf is home to a diverse array of marine organisms that have yet to be fully explored for their biomedical potential. In particular, the sea urchin (*Echinometra mathaei*) and the brittle star (*Ophiocoma erinaceus*) are two echinoderms that are known to possess venom with potential bioactive compounds (Nagle and Paul, 1998; Amini et al., 2015). Some sea urchin species are eaten by locals. Symptoms of intoxication especially after the ingestion of gonads during the breeding season, include allergies as the first signs, nausea, diarrhea, vomiting, upset stomach, severe headaches, swelling of the lips and mouth, salivation, abdominal pain, and some systemic symptoms such as hypotension, numbness, and weakness (Nabipour, 2012). Recent studies have shown that a sea urchin pigment known as spinochromes has cytotoxic activity against the human prostate (Dyshlovoy et al., 2020) and neuro-2a neuroblastoma cancer cells in mice (Polonik et al., 2020). It has been found to have several biological effects, including antiviral activity against HSV-1 infection at various stages of Vero cells (Mishchenko et al., 2020). Sea urchin quinone pigments, especially equinochrome and spinochrome, are known to have potent antioxidant, antibacterial, antifungal, and anticancer properties (Ageenko et al., 2014). The brittle star belongs to the subclass Ophiuroidea, and its name is derived from the Greek words “ophis” meaning snake, and “oura” meaning tail. Various physical defenses have been introduced, such as rapid movement and camouflage under rocks and holes, but some species still rely on chemical defenses. However, so far there are few biological and toxicological studies focused on brittle stars based on the MarineLit database (Kamyab et al., 2020). Various secondary metabolites such as steroids, terpenes, carotenoids (Kamyab et al., 2020), gangliosides, brominated indoles, phenylpropanoids (Nuzzo et al., 2017), and sulfated steroids (Levin et al., 2007; Stonik et al., 2008) have isolated from the brittle stars. A steroidal compound from the brittle star *Ophiocnenmis mormorata* has been shown to have antibacterial, hemolytic, and cytotoxic activities, as well as a disulfated polyhydroxysteroid from the Pacific brittle star *Ophiopolis aculate* as a potent Ca^2+^ agonist in mammalian cell systems (Prabhu and Bragadeeswaran, 2013).

In this study, we aim to uncover the biomedical potential of four venoms derived from these two species, specifically focusing on their antioxidant and anticholinesterase activities. These activities are of particular interest due to their potential therapeutic applications in the treatment of neurodegenerative disorder such as Alzheimer’s disease. By investigating the venom of these sea creatures, we hope to represent a unique contribution to the field of marine biotechnology and highlight the importance of exploring the vast potential of marine organisms for biomedical research.

## 2 Materials and methods

### 2.1 Chemicals

All chemicals, solvents, and standards used for sample extraction and analysis were obtained from chemical companies Merck (Darmstadt, Germany) and Sigma (Deisenhofen, Germany).

### 2.2 Ethical statement

This study was approved by the Medical Ethics Committee of Shiraz University of Medical Sciences and Health Services, Shiraz, Iran. This project was collaborative research with the Bushehr University of Medical in Iran. All animal experiments were conducted according to the National Ethical Guidelines for Iranian Animal Research (2005) under a project license (IR.SUMS.REC.1400.751) approved by the Animal Care and Use Committee of the Shiraz University of Medical Sciences, Iran. Animals were provided with adequate water and food. They were stored under controlled light, temperature, and humidity conditions.

### 2.3 Sampling and sample preparation

**FIGURE 1:**
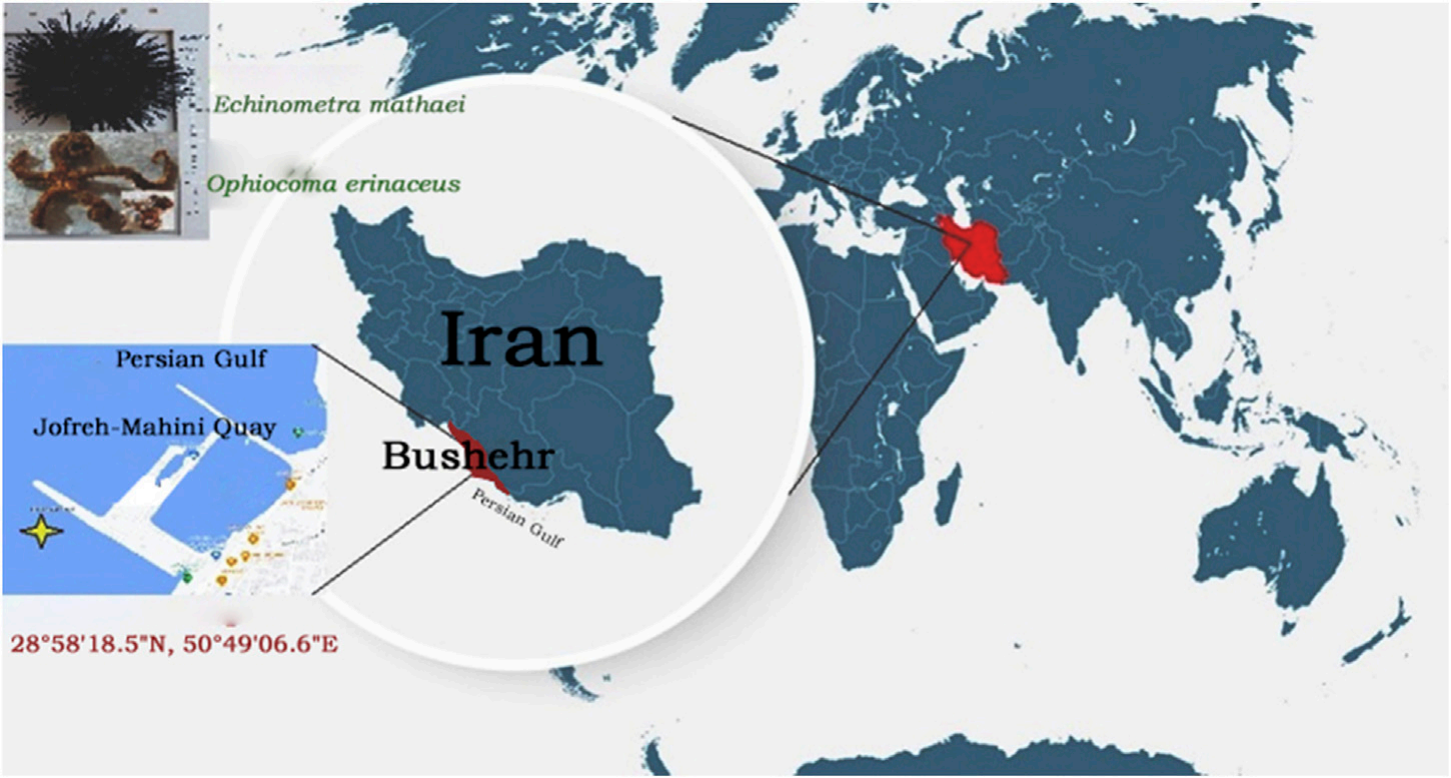
Location map of the sampling area of Brittle Star *O. erinaceus*, and Sea Urchin *E. mathaei* (Jofreh-Mahini Quay waters (28°58′18.5″N, 50°49′06.6″E), Bushehr, Iran).

First, 20 live brittle star *O. erinaceus*, and 20 live sea urchin *E. mathaei* were collected from shallow to deep water at Jofreh-Mahini Quay, Bushehr-Iran (28°58′18.5″N, 50°49′06.6″E), Spring 2021 (Figure 1).

Samples were first washed with seawater to remove impurities and contaminants and stored individually in polypropylene bags with ether-soaked cotton on dry ice, and accordingly conveyed to the laboratories of the Persian Gulf Marine Biotechnology Research Center, which is a part of the Persian Gulf Biomedical Research Center affiliated with Bushehr University of Medical Sciences in Iran. Upon arrival, the samples were promptly frozen at −70°C and kept in storage until they were needed for further analysis. The species of brittle stars and sea urchins were confirmed by morphometric studies by marine biologist Dr. A. Vazirizadeh from the Research Center of Marine Biology, Persian Gulf University, Bushehr, Iran. The Spine Sea Urchin (SSU), Gonad Sea Urchin (GSU), and Brittle Star (BS) were manually isolated from specimens and cut into small pieces with scissors. The Coelomic Sea Urchin (CSU) was also taken using a medical syringe. Following isolation, 100 mg of SSU, GSU, and BS, along with 100 mL of CSU, were individually homogenized using a high-quality homogenizer (IKA, Germany). The homogenates were then subjected to centrifugation at 12,000 × g (Eppendorf, Germany) for 15 min at 4°C to eliminate any sediment. The resulting supernatants, as well as the minute pieces of sea urchin spines and brittle stars, were separated and lyophilized individually using a freeze dryer (Christ, UK). These lyophilized samples were stored at −80°C until they were ready for analysis (Mohebbi et al., 2018).

### 2.4 The median lethal dose (LD_50_)

LD_50_ of the lyophilized gonad, spine, and coelomic fluid of the sea urchin and whole-body brittle star samples dissolved in saline were determined by injecting 0.5 mL appropriate dilutions of the samples (100, 200, 400 μg/mL, and saline as a control group) intravenously into the tail vein of 180 ± 2 g male rats (Four rats per dilution). Mortality was evaluated within 24 h, according to the method of Spearman-Karber (1978), and the results were expressed as (µg/kg) of animal body weight (Spearman-Karber, 1978).

### 2.5 Protein concentration

The total proteins of the four freeze-dried crude venoms were determined by Lowry et al. (1951), using a serially diluted bovine serum albumin (BSA) as standard (200, 400, 600, 800, and 1,000 μg/mL), and then measuring of the absorbances at 660 nm by a spectrophotometer microplate reader (Pharmacia Biotech, Ultra Spect 2000; United States) (Lowry et al., 1951).

### 2.6 Molecular weight determination by SDS-PAGE electrophoresis

A total of 10 μL of each lyophilized venoms (0.1% (w/v)) was subjected to sodium dodecyl sulfate-polyacrylamide gel electrophoresis (SDS-PAGE) at 25°C, according to the Laemmli method (Laemmli, 1970), the following conditions apply: Resolving gel (12.5%, pH: 8); Acrylamide: Bisacrylamide (30:0.8 w/v; 3.1 mL); Tris-HCl (3 mL; 1M; pH: 8.8); SDS (20%, 38 µL); deionized water (1.3 mL); Ammonium persulfate (APS) (10%; 5 µL), and tetram-ethylethylenediamine (TEMED) (10%, 7.5 mL); as well, stacking gel (6%; pH: 6.8): Acrylamide: Bisacrylamide (30: 0.8% (w/v), 1 mL); Tris-HCl (630 μL, 1 M, pH: 8.8); SDS (25 μL; 20%); deionized water (3.6 mL); APS (10%, 24 µL), and TEMED (10%; 5 mL) (Brinkman and Burnell, 2008). The gel was then stained with 0.2% Coomassie Brilliant Blue R-250 (Sigma, Germany) solution in methanol and 25 mL glacial acetic acid in deionized water; then de-staining with 1 L of methanol: glacial acetic acid: H_2_O (2:1:7) until visualizing the protein bands. Molecular weights were estimated in the range of 11–245 kDa by comparison with Sinagen markers (Sinagen, Iran).

### 2.7 Cholinesterases inhibitory activities *in vitro*

The AChE and BChE inhibitory activities of the lyophilized brittle star and sea urchin gonad, spine and coelomic venoms (0.010 mL; 1 mg/mL) were determined at 25°C according to the modified Ellman kinetic method (Worek et al., 1999). The assay is based on the hydrolysis of acetylthiocholine and the formation of thiocholine in the presence of the enzyme. Reduction of the 5, 5′-dithio-bis-2-nitrobenzoic acid (DTNB, Ellman’s reagent) to the yellow anion of 5-thio-2-thionitrobenzoate (TNB^−^) (ε = 10.6×10^3^ mM^-1^Cm^−1^) by thiocholine is measured. Briefly, for AChE and BChE activity, 2,000 mL and 3,000 mL phosphate buffer (0.1 mol/L, pH 7.4), an equal volume of DTNB 0.100 mL (10 mmol/L) for each enzyme, and Ethopropazine 0.010 mL (6 mmol/L) (selective BChEI; used for AChE activity only) were mixed in separate tubes, respectively. The mixture was then incubated for 10 min before adding substrate to allow a complete reaction between the sulfhydryl groups of the sample matrix and DTNB. Afterward, 0.050 mL (28.4 mmol/L) ASCh and 0.050 mL (63.2 mmol/L) BSCh were added to the mixture and recorded the absorbance at 436 nm for 3 min at 30 s intervals using the Cecil UV-VIS spectrophotometer. Then, 60, 200, and 200 μM of Galantamine, AChE, and BChE were added, respectively. The buffer was used in place of blank enzyme solutions. Enzyme activity (Ea) was measured using the following equation:

**I.** Ea μmol/l/min=∆A10.6

**II.** ${∆A=A}_{\text{sample}}\left(mE/\min\right)-A_{\text{Blank}}\;\left(mE/\min\right)$

Percent inhibition (I %) was calculated as:

**III.** $I\;\left(\%\right)=\frac{\left({Ea}_{1}\;-\;{Ea}_{2}\right)}{{Ea}_{1}}\times 100$

Where, “*Ea*_*1*_” is the enzymatic activity without the test compound, and “*Ea*_*2*_” is the enzymatic activity with the test compound. The experiment was performed three times and the mean value was reported. The concentration of the sample that inhibited substrate hydrolysis by 50% (IC_50_) was determined by linear regression analysis between percentage inhibition and sample concentration using an Excel program.

### 2.8 GC-MS analysis of the crude venoms

All four lyophilized crude venoms were subjected to a 7890B Agilent Gas Chromatography Mass Spectrometry (Agilent GC-MS, Germany), after sample extraction with methanol: chloroform: n-Hexane (2:2:1 v/v), aiming to achieve their chemical compositions. Mass spectra were taken at ionization energy of 70 eV, filament emission of 0.5 mA, scan interval of 0.5 s, and fragments from m/z 50–500 Da. GC separation was performed using an HP-5MS UI capillary column (30 m × 0.25 mm ID × 0.25 μm i. d, and 0.5 μm film thickness). Helium was used as the carrier gas at constant flow rate of 0.8 mL min^-1^, injection volume of 1 μL, and split ratio (30:1). The injection port, transfer line, and ion source temperatures were maintained at 240°C, 250°C, and 270°C, respectively. The oven temperature was programmed from 80°C after 3 min to 250°C at a rate of 5°C per minute and held for 10 min. The total GC run time was 37 min. The isolated compounds were identified by comparing them with data from the National Institute of Standards and Technology Library (NIST MS database, 2014). The relative quantity (%) of each component was determined by comparing the average peak area with the total area (Zare et al., 2023).

### 2.9 Computational details

All recognized compounds and galantamine, utilized as a reference standard, underwent testing for their inhibitory effects on AChE and BChE. The compounds were energy minimized using the DFT method and the b3lyp/6–311g basis set with the Gaussian09 program (Gaussian et al., 2009). These optimized compound structures were then employed in docking studies against AChE (PDB code: 4BDT) and BChE (PDB code: 4TPK) using Autodock Vina Software (Maryamabadi et al., 2016).

### 2.10 Antioxidant activity

The antioxidant activity (AA; %) of four lyophilized SSU, GSU, CSU, and BS venoms was performed using the DPPH scavenger method according to Marhamati et al. (Marhamati et al., 2021). First, 200 mg of each lyophilized sample with different concentrations ranging from 50 to 200 μg/mL were mixed with 800 μL of methanol. 400 μL of each diluted sample was then homogenized with 1.6 mL of DPPH solution (0.1 mM). The solution was kept in the dark at 25°C for 1 h and finally, the absorbance at 517 nm was measured. Free lyophilized sample was used as control group. Inhibitory activity (%) was obtained from the following formula:

$$I\;\left(\%\right)=\frac{A_{c}-\;A_{s}}{A_{c}}\times 100$$

Where, *A*_*c*_: Absorbance of control; *A*_*s*_ Absorbance of the sample.

### 2.11 Statistical analyses

Statistical analysis was performed with SPSS for Windows V.24, and Excel software was for graph drawing. All data were expressed as the mean ± SD for each group. Differences between individual groups were analyzed by one-way analysis of variance test (ANOVA) and then by Duncan’s test. *p* < 0.05 was considered statistically significant.

## 3 Results

### 3.1 LD_50_

The LD_50__(IV rat)_ values for the spine, gonad, and coelomic fluid of *Echinometra mateii* were respectively estimated at 2.231 ± 0.09, 1.03 ± 0.05, and 1.12 ± 0.13 mg/kg BW in a 24 h observation period, as well as 6.04 ± 0.13 mg/kg BW for *O. erinaceus*. According to the statistical analysis, excepting the gonad and coelomic fluid samples (*p*: 0.325), the LD_50_ levels were significantly different between other groups (*p* < 0.05).

### 3.2 The total proteins concentration and SDS-PAGE analysis

**FIGURE 2:**
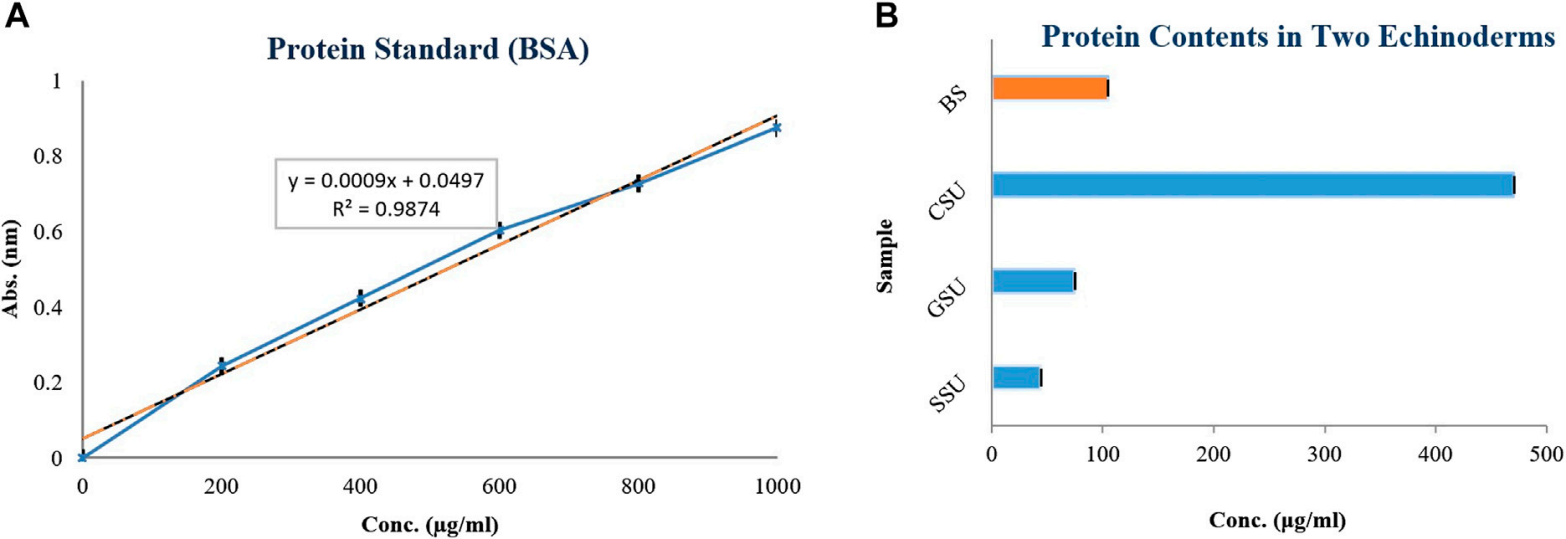
Calibration curve of bovine serum albumin (**BSA**) at concentrations of 200, 400, 600, 800, and 1,000 μg/mL, at 660 nm **(A)**; The total proteins of four lyophilized crude venoms Spine Sea Urchin (**SSU**), Gonad Sea Urchin (**GSU**), Coelomic Sea Urchin (**CSU**), and Brittle Star (**BS**) (as µg/mL) **(B)**.

Total protein at 660 nm by BSA standard for the four lyophilized crude venoms SSU, GSU, CSU, and BS was 44.037 ± 0.002, 74.223 ± 0.025, 469.97 ± 0.02 and 104.407 ± 0.025 μg/mL, respectively. As shown in Figure 2, the highest total protein content among sea urchin samples was associated with coelomic fluid, followed by gonad and spine. All group differences were significant (*p* < 0.05). The average total protein content of sea urchin samples (196.076 μg/mL) was also significantly higher than that of the brittle star.

**FIGURE 3:**
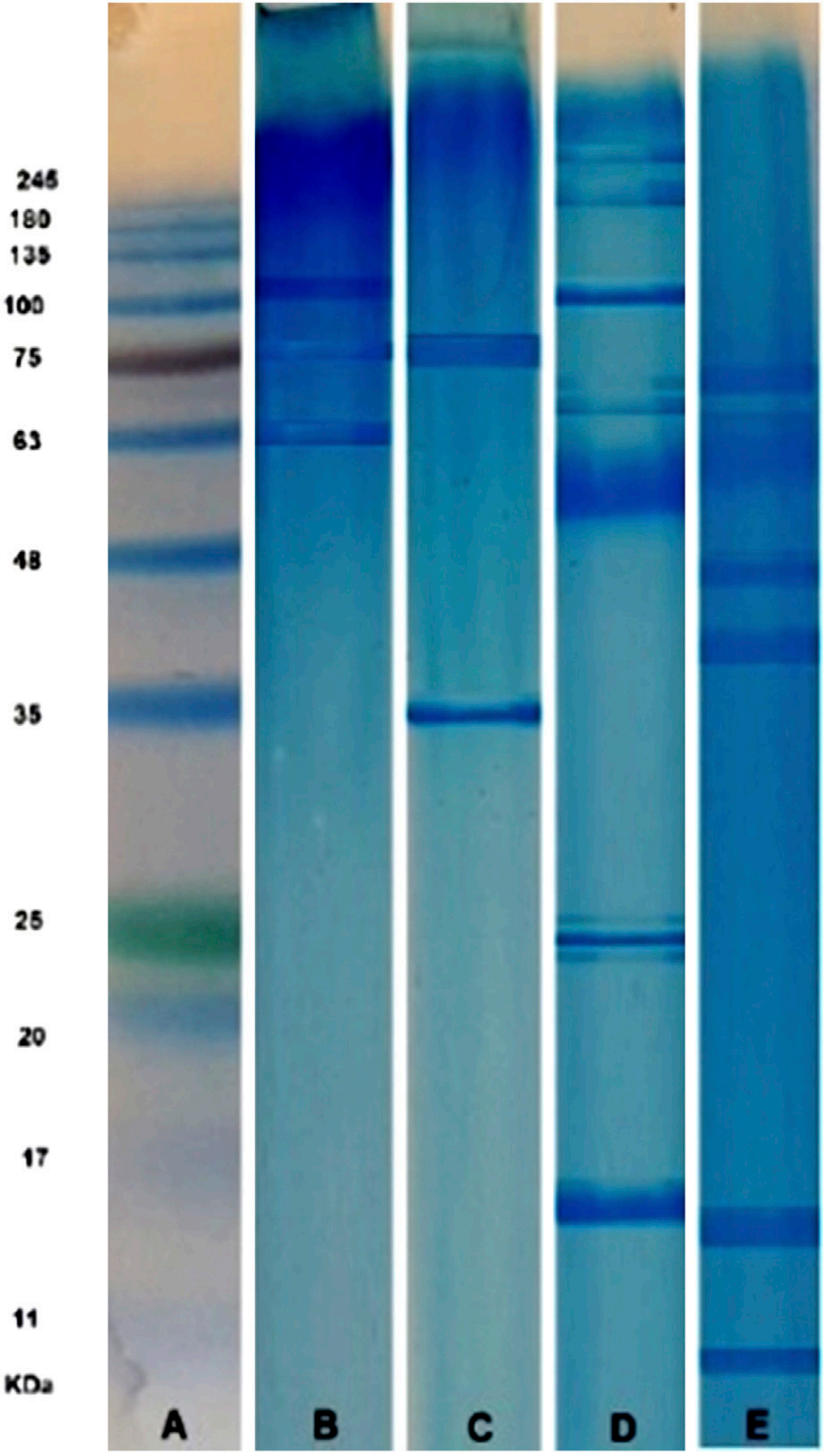
SDS-PAGE analysis (12% polyacrylamide gel stained with Coomassie brilliant blue) of SinaGen protein standard marker **(A)**, and four Gonad Sea Urchin **(B)**, Coelomic Sea Urchin **(C)**, Spine Sea Urchin **(D)**, and Brittle Star **(E)** lyophilized crude venom samples.

Compared to the protein standard markers (Figure 3A), SDS-PAGE analysis of GSU sample revealed two prominent bands with masses between 63 and 75 kDa and one band between 100 and 135 kDa (Figure 3B); In addition, CSU extract revealed two distinct bands at approximately 35 kDa and 75 kDa (Figure 3C). Furthermore, the SSU sample had at least 10 prominent protein bands with molecular weights ranging from 17 to 245 kDa. Major bands were found at putative molecular weights of 17, 25, 100, 180, and 225 kDa, along with two other prominent bands at 63–75 kDa, as well as an intense band in the 48–63 kDa range. Besides, two other bands were also detected near the 25 KDa bands (Figure 3D). In addition, SDS-PAGE analysis of BS revealed at least six prominent protein bands with molecular weights ranging from 11 to 75 kDa. Major bands were located at approximately molecular weights 11, 48, and 63, with three major bands between regions 11–17, 35–48, and 63–75 kDa (Figure 3).

### 3.3 Anticholinesterase activities

A total of four lyophilized venoms SSU, GSU, CSU, and BS were screened for *in vitro* AChE and BChE inhibitory activity. Ellman’s spectroscopy-based experimental data revealed the appropriate ChE inhibitory activity for each of four venoms studied. As shown in Table 1, GSU exhibited the most promising AChEI activity with IC_50_ value of 9.302 ± 0.001 μM, followed by inhibitory properties of CSU, SSU, and BS with IC_50_ values of 16.658 ± 0.003, 29.161 ± 0.08 and 37.925 ± 0.055 μM compared to galantamine (9.307 ± 0.126 μM). Regarding statistical analysis, although there was no significant difference between the sea urchin gonad sample and the galantamine standard (*p* = 0.905), the AChE IC_50_ was significantly different between all other samples and the galantamine standard (*p* < 0.05). Moreover, they had some degrees of inhibitory activity against BChE. BS lyophilized skeleton was found to be most active at IC_50_: 5.388 ± 0.02 μM; then GSU, SSU, and CSU, with IC_50_ values of 10.056 ± 0.285, 25.983 ± 0.003, and 62.627 ± 0.14 μM, respectively, compared to galantamine (9.367 ± 0.031 μM). The IC_50_ (μM) and selectivity values of the samples against AChE and BChE are summarized in Table (1). Based on statistical analysis, the IC_50_ of the BChE enzyme, all samples were significantly different from each other and the galantamine standard (*p* < 0.05).

**TABLE 1:** Acetyl and butyrylcholinesterase inhibitory activity of four SSU, GSU, CSU, and BS lyophilized venom compounds.

Sample	IC_50_ (μM)		Selectivity^a^	
	AChE	BChE	AChE	BChE
**SSU**	29.161 ± 0.08	25.983 ± 0.003	0.891	1.122
**GSU**	9.302 ± 0.001	10.056 ± 0.285	1.081	0.925
**CSU**	16.657 ± 0.003	62.627 ± 0.14	3.759	0.265
**BS**	37.925 ± 0.55	5.388 ± 0.02	0.142	7.038
**Galantamine**	9.307 ± 0.126	9.367 ± 0.031	1.006	0.993

^a^ Selectivity for AChE is defined as IC_50_ (BChE)/IC_50_ (AChE) and Selectivity for BChE is defined as IC_50_ (AChE)/IC_50_ (BChE).

### 3.4 GC-MS analysis of the crude venoms

Analysis of the SSU venom using the GC-MS, has detected twelve compounds (**S**_**1**_-**S**_**12**_) with the retention time (RT), 6.905, 8.908, 11.898, 11.998, 12.77, 17.411, 17.429, 17.503, 22.755, 22.771, 29.601, and 35.789 min, respectively. Patterns were consistent with **S**_**1**_: 2-Propenamide; (C_3_H_5_NO (0.245%)); MW: 71.077 (g/mol)., **S**_**2**_: Tridecane; (C_13_H_28_ (0.184%)); MW: 184.36., **S**_**3**_: Pyridine, 4-(4-methyl-5-trans-phenyl-1,3-oxazolidin-2-yl); (C_15_H_16_N_2_O (0.241%)); MW: 240.30., **S**_**4**_: 2,2,6,6, -Tetramethyl-4-acetoxyimino piperidine; (C_11_H_20_N_2_O_2_ (0.163%)); MW: 212.29., **S**_**5**_: Harmine; (C_13_H_12_N_2_O (0.294%)); MW: 212.25., **S**_**6**_: Decanoic acid, 2-methyl-; (C_11_H_22_O_2_ (1.056%)); MW: 186.29., **S**_**7**_: 9-Hexadecenoic acid, methyl ester, (z)-; (C_17_H_32_O_2_ (7.382%)); MW: 268.4. **S**_**8**_: 2-o-methyl-d-xylose; (C_6_H_12_O_5_ (0.348%)); MW: 164.16., **S**_**9**_: 9,12-octadecadienoic acid (z, z)-, methyl ester; (C_19_H_34_O_2_ (0.167%)); MW 294.47., **S**_**10**_: Octadecanoic acid, methyl ester; (C_19_H_38_O_2_ (0.898%)); MW: 298.5., **S**_**11**_: Astaxanthin; (C_40_H_52_O_4_ (27.100%)); MW: 596.8., **S**_**12**_ **:** Cholesterol; (C_27_H_46_O (386.7%)); MW: 61.921. These height and low molecular weight molecules belong to different groups of amides, alkaloids, terpenoids, alkanes, fatty acids, esters, and steroids, which are considered biologically and pharmacologically important.

The GC-MS study of GSU extract revealed a wide variety of phytochemicals (**G**_**1**_-**G**_**23**_) including as alkaloids, steroids, monoterpenoids, diterpenes, sesquiterpenes, fatty acid esters, amides, benzopyrans, alkenes, carbohydrates, vitamins, and alcohols (Table 2).

**TABLE 2:** The GC-MS analysis of GSU extract indicated 23 phytochemicals (G_1_-G_23_), with different chemical and bioactive structures.

No.	Compound	Molecular formula	MW (g/mol)	Abundance (%)	RT (min)
**G**_**1**_	2,5-Dimethyl-1-pyrroline	C_6_H_11_N	97.158	1.298	6.103
**G**_**2**_	3,3-Diphenyl-5-methyl-3H-pyrazole	C_16_H_14_N_2_	234.29	0.466	9.184
**G**_**3**_	Tetradecanoic acid, 10, 13-dimethyl-, methyl ester	C_17_H_34_O_2_	270.5	0.142	12.740
**G**_**4**_	N-(1-Naphthyl) lauramide	C_22_H_31_NO	325.48	0.396	13.648
**G**_**5**_	Goitrin	C_5_H_7_NOS	129.18	0.256	13.704
**G**_**6**_	Citrinin	C_13_H_14_O_5_	250.25	1.650	17.436
**G**_**7**_	1-Phenazine carboxylic acid, 6[1-[(1-oxohexadecyl) oxy) ethyl]-	C_31_H_42_N_2_O_4_	506.7	2.119	18.519
**G**_**8**_	1,8-Nonadiene,2-methyl-5,7-dimethylene-	C_12_H_18_	162.27	1.217	25.538
**G**_**9**_	à-Farnesene	C_15_H_24_	204.35	0.838	25.667
**G**_**10**_	4,6-O-Furylidene-d-glucopyranose	C_11_H_14_O_7_	258.22	0.358	25.877
**G**_**11**_	1, E-8, Z-10-Pentadecatriene	C_15_H_26_	206.37	0.634	25.982
**G**_**12**_	1H-Tetrazole, 1-methy-	C_2_H_4_N_4_	84.08	0.351	26.269
**G**_**13**_	2-Piperidinone, N-[4-bromo-n-butyl]-	C_9_H_16_BrNO	234.13	0.339	27.732
**G**_**14**_	Bacchotricuneatin C	C_20_H_22_O_5_	342.4	0.431	28.867
**G**_**15**_	1-Phenyl-3,5,7-trimethyl-6,7(8H)-dihydropyrazolo(3,4-b) (1,4) diazepine	C_15_H_18_N_4_	254.33	1.319	29.236
**G**_**16**_	Aspidofractinine-3-methanol, (2à, 3à,5à)-	C_20_H_26_N_2_O	310.4	4.059	29.608
**G**_**17**_	D-Mannitol,1-decylsulfonyl-	C_16_H_34_O_7_S	370.5	0.629	29.792
**G**_**18**_	Quinoline, 8-bromo-	C_9_H_6_BrN	208.05	2.126	30.279
**G**_**19**_	Androst-11-en-17-one,3-formyloxy-, (3à, 5à)-	C_20_H_28_O_3_	316.4	12.458	31.368
**G**_**20**_	Spirost-8-en-11-one, 3-hydroxy-, (3à, 5à, 14à, 20à, 22à, 25R)-	C_27_H_40_O_4_	428.6	2.529	33.158
**G**_**21**_	Sarpagan-17-ol, 16-[(acetyloxy) methyl]-, acetate (ester)	C_24_H_28_N_2_O_4_	408.5	13.599	33.720
**G**_**22**_	Strychane, 1-acetyl-20à-hydroxy-16-methylene-	C_21_H_26_N_2_O_2_	338.4	51.524	35.804
**G**_**23**_	Vitamin E acetate	C_31_H_52_O_3_	472.7	1.263	36.034

The results of phytochemical analysis of CSU were shown in Table 3. The results showed that the sample contains 21 different classes of secondary metabolites (**C**_**1**_-**C**_**21**_), including alkaloids, sterols, alcohols, ketones, esters, organic acids, vitamins, monoterpenes, triterpenes, heterocyclic compounds, furanocoumarins and ethers.

**TABLE 3:** The GC-MS analysis of the CSU, showed 21 compounds (C_1_-C_21_), in different types of phytochemical groups.

No.	Compound	Molecular formula	MW (g/mol)	Abundance (%)	RT (min)
**C**_**1**_	1,5- Pentanediol, 3-methyl-	C_6_H_14_O_2_	118.17	0.637	6.205
**C**_**2**_	4-Octanone	C_8_H_16_O	128.21	0.182	7.772
**C**_**3**_	3,3-Diphenyl-5-methyl-3H-pyrazole	C_16_H_14_N_2_	234.29	0.621	9.188
**C**_**4**_	Cyclobuta[b]quinoline-8-carboxylic acid, 1,2-dihydro-	C_12_H_9_NO_2_	199.20	0.158	12.719
**C**_**5**_	Isoxazolidine, 5-ethyl-2,4-dimethyl-trans-	C_7_H_15_NO	129.20	0.422	13.640
**C**_**6**_	Hexadecanoic acid, methyl ester	C_17_H_34_O_2_	270.5	1.606	17.422
**C**_**7**_	6H-Indolo[3,2,1-de] [1,5] naphthyridine-6-one,1,2,3a,4,5-hexahydro-8-hydroxy-3-methyl	C_15_H_16_N_2_O_2_	256.30	2.462	18.513
**C**_**8**_	Tetradecanoic acid, 12-methyl-, methyl ester, (s)-	C_16_H_32_O_2_	256.42	0.345	22.765
**C**_**9**_	1,3,6-Octatriene, 3,7-dimethyl-, (E)-	C_10_H_16_	136.23	0.668	25.536
**C**_**10**_	Cyclohexanecarboxaldehyde,4-(hydroxymethyl)-	C_8_H_14_O_2_	142.20	0.321	25.864
**C**_**11**_	Ethanamine,2-[(4-chlorophenyl)-2-pyridinylmethoxy]-N, N-dimethyl-	C_16_H_19_ClN_2_O	290.79	0.324	25.979
**C**_**12**_	1-Hexyl-2-nitrocyclohexane	C_12_H_23_NO_2_	213.32	0.269	26.280
**C**_**13**_	2-Piperidinone, N-[4-bromo-n-butyl]-	C_9_H_16_BrNO	234.13	0.175	27.725
**C**_**14**_	Cyclohexanecarboxamide, N-furfuryl-	C_12_H_17_NO_2_	207.27	1.062	29.055
**C**_**15**_	Cyclopenta[c] furo [3ˊ, 2ˊ:4,5] furo [2,3-h] [1] benzopyran-11(1H)-one, 2, 3,6a, 9a-tetrahydro-1,3-dihydroxy-4-methoxy-	C_17_H_14_O_7_	330.29	3.052	29.216
**C**_**16**_	Astaxanthin	C_40_H_52_O_4_	596.8	4.843	29.601
**C**_**17**_	3-methyl-4-(methoxycarbonyl) hexa-2, 4-dienoic acid	C_9_H_12_O_4_	184.19	0.639	29.798
**C**_**18**_	Octadecane, 1-(ethenyloxy)-	C_20_H_40_O	296.5	2.206	30.252
**C**_**19**_	Squalene	C_30_H_50_	410.7	2.643	32.476
**C**_**20**_	Cholesterol	C_27_H_46_O	386.7	75.714	35.804
**C**_**21**_	Vitamin E	C_29_H_50_O_2_	430.7	1.653	36.030

Phytochemical screening of lyophilized whole **BS** by GC-MS method revealed the presence of 25 bioactive components (**BS**_**1**_- **BS**_**25**_) (Table 4).

**TABLE 4:** Phytochemical screening of the lyophilized BS by GC-MS method revealed the presence of 25 bioactive compounds (**BS**_**1**_- **BS**_**25**_).

No.	Compound	Molecular formula	MW (g/mol)	Abundance (%)	RT (min)
**BS**_**1**_	1-Pentanamine, N-nitro-	C_5_H_12_N_2_O_2_	132.16	0.018	6.814
**BS**_**2**_	2-Bromononane	C_9_H_19_Br	207.15	0.023	7.779
**BS**_**3**_	Decane,2,4,6-trimethyl-	C_13_H_28_	184.36	0.032	8.868
**BS**_**4**_	(3H) Pyrazole,3,5-diphenyl-3-methyl-	C_16_H_14_N_2_	234.29	0.208	9.186
**BS**_**5**_	1H-Tetrazol-5-amine	CH_3_N_5_	85.07	0.010	9.468
**BS**_**6**_	Squalene	C_30_H_50_	410.7	0.031	16.065
**BS**_**7**_	Anosmagenin	C_27_H_42_O_5_	446.61	0.023	17.324
**BS**_**8**_	n-Hexadecanoic acid	C_16_H_32_O_2_	256.42	0.144	17.435
**BS**_**9**_	Ethaneperoxoic acid,1-cyano-1-[2-(-phenyl-1,3-dioxolan-2-yl) ethyl] penthyl ester	C_19_H_25_NO_5_	347.4	0.189	18.508
**BS**_**10**_	3H-1,2,4-Triazole-3-thione,2,4-dihydro-2,4,5-trimethyl-	C_5_H_9_N_3_S	143.21	0.061	22.775
**BS**_**11**_	Octadecanoic acid	C_18_H_36_O_2_	284.5	0.078	23.790
**BS**_**12**_	Ergotaman-3ˊ,6ˊ,18-trione,12ˊ-hydroxy-2ˊ-methyl-5ˊ-(2-methylpropyl)-, (5ˊà)-	C_30_H_37_N_5_O_5_	547.6	0.200	22.723
**BS**_**13**_	Pseduo sarsasapogenin-5,20-dien	C27H42O3	414.6	1.150	29.609
**BS**_**14**_	Spirost-8-en-11-one, 3-hydroxy-, (3à, 5à, 14à, 20à,22à, 25R)-	C_27_H_40_O_4_	428.6	5.667	31.197
**BS**_**15**_	Pregnane-3,11,20,21-tetrol, cyclic 20,21- (butylboronate), (3à, 5à,11à, 20R)-	C_25_H_43_BO_4_	418.4	12.933	31.491
**BS**_**16**_	Megestrol Acetate	C_24_H_32_O_4_	384.5	73.679	32.025
**BS**_**17**_	Ergosta-5,22-dien-3-ol, acetate, (3à, 22E)-	C_30_H_48_O_2_	440.7	0.233	32.175
**BS**_**18**_	5-(P-Aminophenyl) -4-(p-tolyl) -2-thiazolamine	C_16_H_15_N_3_S	281.4	0.460	32.480
**BS**_**19**_	5-Pregnen-3à, 9à-diol-20-one 3-acetate	C_23_H_34_O_4_	374.5	0.878	32.775
**BS**_**20**_	Cholesta-3,5-diene	C_27_H_44_	368.6	0.654	33.155
**BS**_**21**_	Sarpagan-17-ol,16-[(acetyloxy)methyl)-, acetate (ester]	C_24_H_28_N_2_O_4_	408.5	0.580	35.290
**BS**_**22**_	Demecolcine	C_21_H_25_NO_5_	371.4	0.339	35.394
**BS**_**23**_	.psi.,.psi.-Carotene, 1,1′,2,2′-tetrahydro-1,1′-dimethoxy-	C_42_H_64_O_2_	601.0	1.130	35.792
**BS**_**24**_	à-Hydroxy quebrachamine	C_19_H_26_N_2_O	298.4	0.901	36.468
**BS**_**25**_	Cholic acid	C_24_H_40_O_5_	408.6	0.378	37.368

### 3.5 Molecular docking

To better understand the experimental results, molecular docking studies of AChE and BChE were performed on the identified biomolecules. Table 5 shows the estimated binding energies of the 81 identified compounds in the four lyophilized SSU, GSU, CSU, and BS toxins.

**TABLE 5:** The estimated binding energy from molecular docking study of the 81 identified compounds in four SSU, GSU, CSU, and BS lyophilized venoms.

compound No	Affinity (kcal/mol)							
	AChE				BChE			
*	BS	G	C	S	BS	G	C	S
1	−5.2	−4.9	−4.9	−4.2	−4.7	−4.1	−4.1	−3.5
2	−5.9	−10.0	−5.6	−6.1	−4.8	−6.9	−4.4	−4.8
3	−6.4	−6.9	−9.9	−8.8	−5.2	−5.6	−7.1	−7.5
4	−10.1	−9.0	−9.3	−7.2	−8.2	−6.9	−7.7	−6.4
5	−4.5	−4.4	−4.8	−9.2	−3.8	−4.2	−4.4	−7.8
6	−8.7	−8.6	−8.6	−6.7	0.9	−7.8	−7.5	−5.6
7	−5.3	−7.5	−10.5	−6.5	21.6	6.3	−8.2	−5.3
8	−6.5	−6.5	−6.8	−5.1	−5.5	−5.4	−5.3	−4.4
9	−8.7	−7.7	−6.8	−7.2	−7.4	−5.8	−5.4	−5.2
10	−5.3	−7.9	−6.3	−6.7	−4.5	−7.0	−5.6	−4.7
11	−6.8	−6.6	−8.9	11.5	−5.5	−5.6	−6.3	173.1
12	−0.7	−4.0	−7.5	−6.3	8.9	−3.4	−6.4	−4.7
13	−2.2	−6.2	−6.1		−0.6	−5.3	−5.3	
14	−5.3	−7.9	−8.1		14.2	−6.7	−6.3	
15	−5.9	−9.0	−9.6		−5.7	−7.3	−6.6	
16	−5.9	−7.9	11.5		−6.3	−7.6	173.1	
17	−2.2	−7.2	−6.3		1.4	−4.6	−5.5	
18	−10.0	−7.6	−6.5		−5.8	−6.2	−4.8	
19	−5.8	−7.9	−8.7		−5.6	−7.4	0.9	
20	−5.7	−5.3	−6.2		−5.0	14.2	−5.3	
21	−8.2	−8.2	−7.1		−5.3	−5.3	0.7	
22	−8.5	−7.0			−5.0	−5.5		
23	6.2	−5.9			112.9	3.2		
24	−8.0				−6.7			
25	−4.0				−3.6			
Gal			−7.1				−5.9	

(S): Spine Sea Urchin, (G) Gonad Sea Urchin, (C): Coelomic Sea Urchin, and (BS): Brittle Star

Among the major bonds of **BS**_**4**_ ligand with AChE enzyme, can be revealed two *Pi-Pi* Stacked bonds between the aromatic group of both amino acids TRP A: 86 and TYRA: 337 with the 3-phenyl group of the compound; Two Hydrogen bonds, one between the amino acid TYRA: 337 and the nitrogen atom (no. 2); and the other between the amino acid TYR A: 124 and the nitrogen atom (no. 1) of the pyrazole nucleus of the compound; as well, the Pi-Alkyl bond between the pyrazole and TRP A: 86. About the major interactions of **G**_**2**_ with the enzyme, in addition to von der Waals bonds of the compound with amino acids such as TYR A: 449; TYR A: 133; GLY A: 121; TYR A: 124, and SER A: 125, it can be referred to the two Pi-Pi Stacked bonds between the amino acids TYR A: 337 and TRP A: 86 with the aromatic group of the compound; an amid Pi Stacked bond between the amino acid GLY A: 126 with the aromatic group of the ligand; as well, two Pi Alkyl bonds between the methyl group of the pyrazole nucleus of the compound with the amino acids HIS A: 447 and PHE A: 338. Of the significant interactions of the **S**_**5**_ compound, the hydrogen bond between the amino acid HIS A: 447 and the hydrogen of the indole ring compound, six Pi-Pi Stacked bonds between three compound rings with the amino acids TYR A: 337 and TRP A: 86, Another Pi-Pi Stacked bond between the amino acid HIS A: 447 and the compound ring, two Pi-Alkyl bonds between the pyridine nucleus methyl group compound with two amino acids TRP A: 439 and TYR A: 449 and another Pi-Alkyl bond. The combination between the amino acid TYR A: 337 and the methyl group of the pyridine nucleus can be mentioned.

**FIGURE 4:**
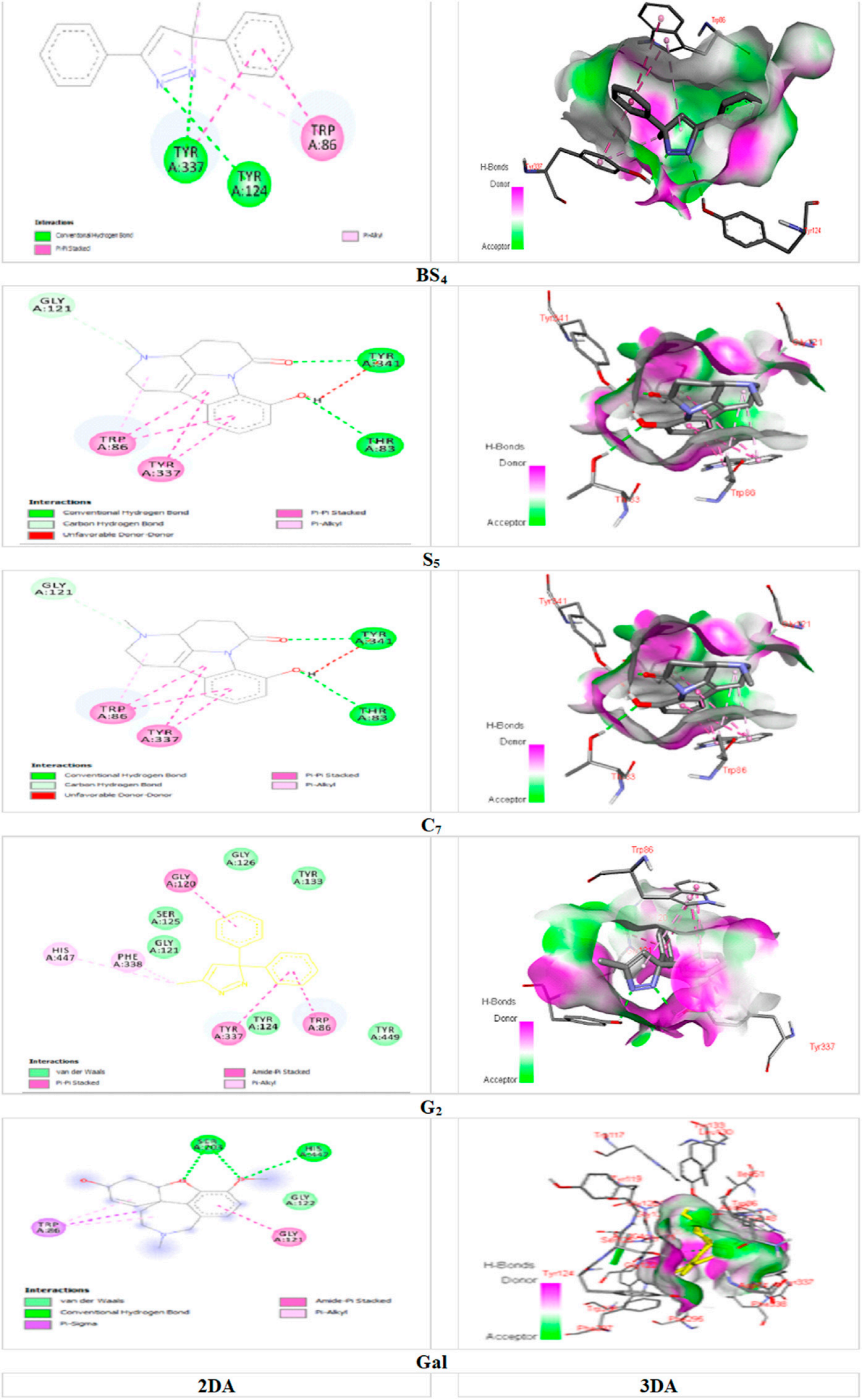
Docking of the isolated compounds (**BS_4_**, **S_5_**, **C_7_**, and **G_2_**) with the highest binding energy, and galantamine (as the standard molecule), with AChE enzyme.

**FIGURE 5:**
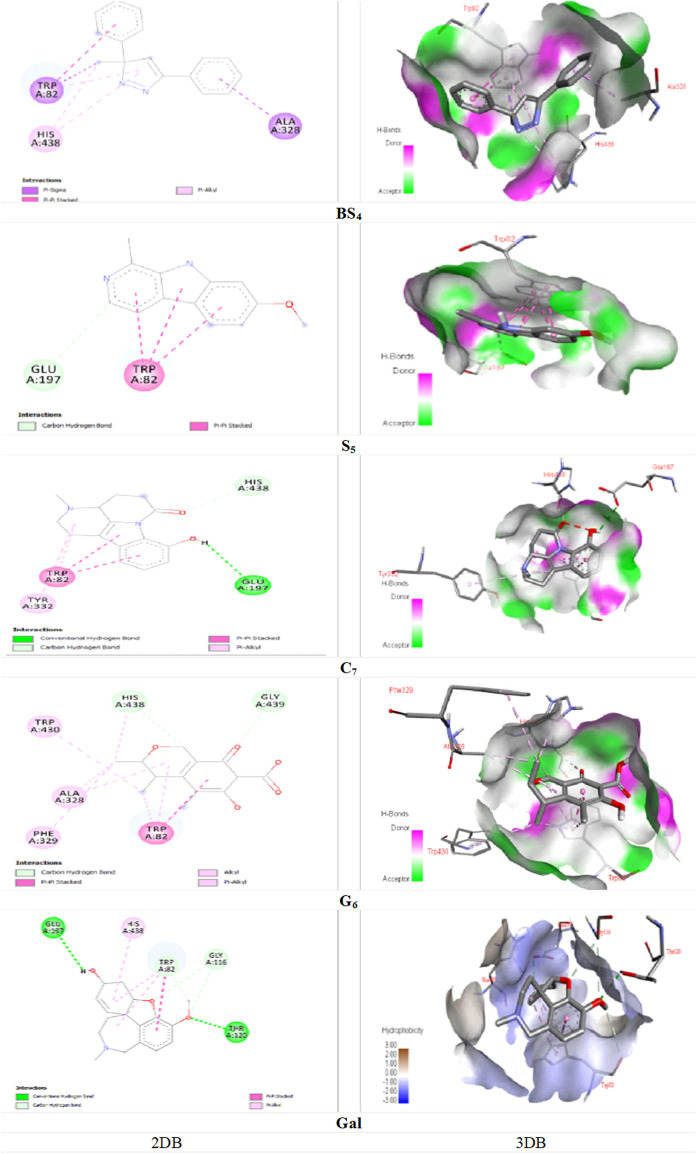
Docking of the isolated compounds (**BS**_**4**_, **S**_**5**_, **C**_**7**_, and **G**_**6**_) with the highest binding energy, and galantamine (as the standard molecule), with BChE enzyme.

Figure 5, demonstrated the docking results of ligands with the highest binding energy to the BChE enzyme. The key interaction between **BS_4_** and the BChE enzyme involves the Pi-sigma bond between the amino acid ALA A: 328 and the 5-phenyl group of the compound; the other Pi-Sigma bond between the amino acid TRP A: 82 and the methyl group of the pyrazole nucleus; a Pi-Pi Stacked bond between this amino acid and the 3-phenyl group of the compound; two π-Alkyl bonds between the methyl group and the pyrazole nucleus of compound containing the amino acid HIS A: 438; as well as other Pi-Alkyl bond between the pyrazole nucleus and the amino acid TRP A: 82. A notable interaction between **S_5_** and BChE involves the C-H bond (carbon-hydrogen) of amino acid GLU A: 197 with the pyridine core of the compound. The same applies to TRP A: 82, has three pi-pi stacking bonds associated with each **S_5_** ring, contributing a remarkable affinity for the bound enzyme. It is noteworthy that the C-H (carbon-hydrogen) bonds between the amino acids HIS A: 438 and GLY A: 439 respectively, with the furan ring and the carbonyl group from cyclohexane ring of the **G**_**6**_; A Pi-Pi Stacked bond between the amino acid TRP A: 82 and the cyclohexanone ring of the ligand; A Pi-Alkyl bond between the amino acid TR A: 82 and the pyran ring; Another Pi-Alkyl bond between ALA A: 328 with pyran ring and Alkyl bonds between amino acids TRP A: 82, ALA A: 328, PHE A: 329, TRP A: 430 and HIS A: 438 with different fragments of the molecule **G_6_** were the major interactions between the ligand and the enzyme. Molecular docking studies of AChE enzymes and ligands showed that the **C_7_** compound was the most active compound against ChE compared to all other compounds as well as the control molecule galantamine. This is likely due to the presence of two hydrogen bonds (Figure 4). A hydrogen bond between amino acid TYR A: 341 and carbonyl group of 1-5 naphthyridine from compound **C_7_**, and another hydrogen bond between amino acid THR A: 83 and hydroxyl group (-OH) of the aromatic ring from indole group of the ligand; an Unfavorable Donor-Donor bond between amino acid TYR A: 341 and (-OH) of the aromatic ring from the indole group of the compound; a C-H (carbon-hydrogen) bond between amino acid GLY A: 121 with methylated nitrogen atom of pyridine nucleus of the compound; two Pi-Pi stacked bonds between the amino acid TYR A: 337 and the aromatic ring of the indole group and the other with the pyrrole ring; two Pi-Pi stacked bonds between amino acid TRP A: 86 with pyrrolic and aromatic rings of the indolic group and a Pi-Alkyl bond between TRP A: 86 and the pyridine nucleus of compound were detected. For the interaction of ligand **C**_**7**_ with the BChE enzyme, a hydrogen bonding between the amino acid GLU A: 197 and the (-OH) of the phenyl group from the indole nucleus of the compound; a C-H bond between the amino acid HIS A: 438 and the carbonyl group of the naphthyridine; in addition, two Pi-Pi Stacked bonds between the amino acid TRP A: 82 with both indole nucleus rings of the compound; A Pi-Alkyl bond between the indicated amino acid and the pyridine ring, and one more between the amino acid TYR A: 332 and the pyridine ring can be revealed. Strong inclusion bonds are formed due to the strength of hydrogen bonding and π-π bonding between the ligand and the enzyme (Figure 5).

### 3.6 Antioxidant activity

**FIGURE 6:**
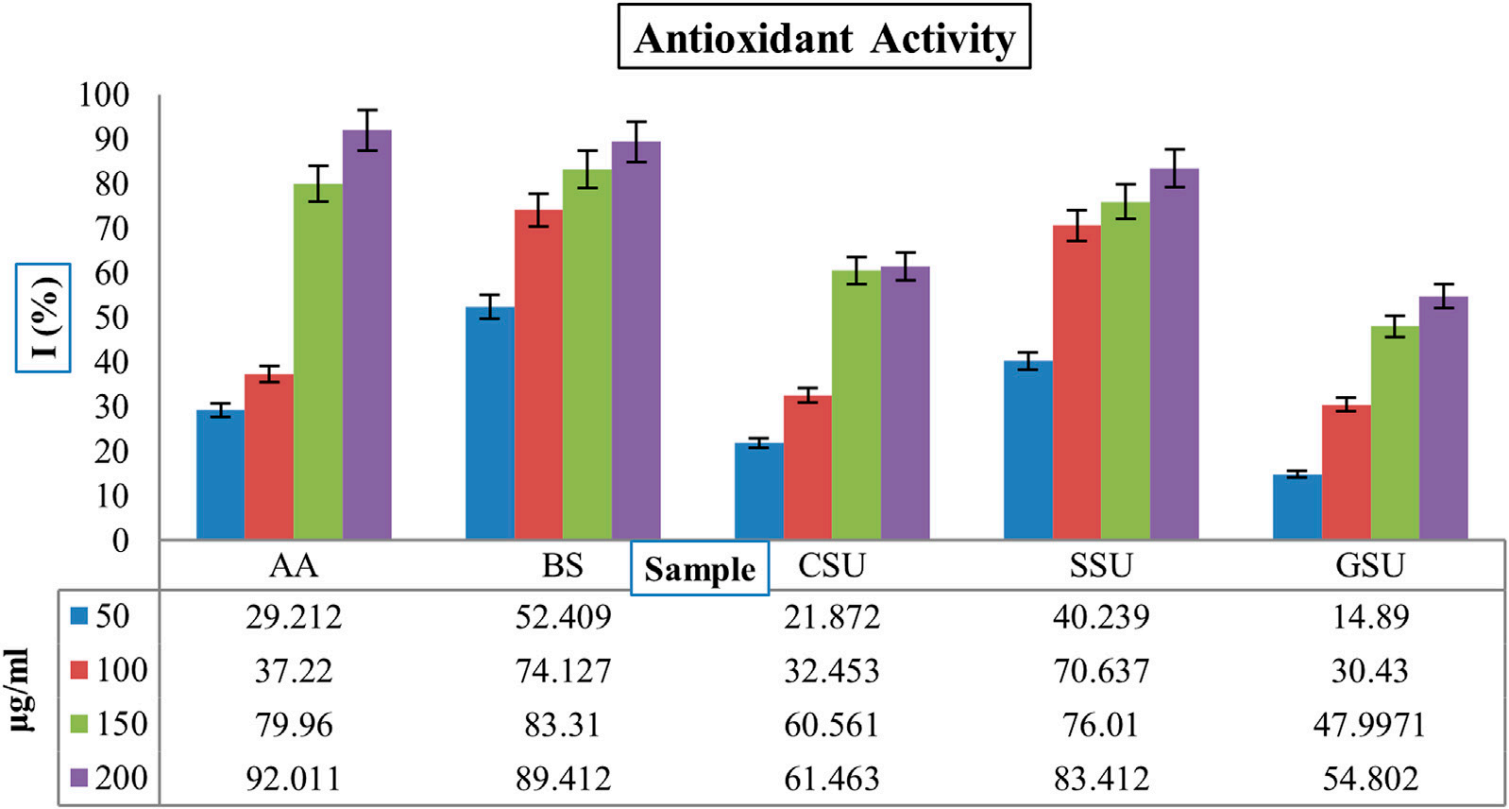
The inhibitory activities (%) of four lyophilized crude venoms SSU, GSU, CSU, and BS through the AA as standard against free radical DPPH. AA: ascorbic acid; SU: Spine Sea Urchin; GSU: Gonad Sea Urchin; CSU: Coelomic Sea Urchin, and BS: Brittle Star.

The Figure 6 shows the inhibitory activity (%) of the four lyophilized crude venoms SSU, GSU, CSU, and BS with ascorbic acid (AA) as standard against free radical DPPH. The results showed that the BS sample exhibited significantly higher antioxidant activity than the other samples, as well as standard ascorbic acid at all concentrations (*p* < 0.05). Dose-dependently, the **BS** sample concentration from 50 μg/mL to 200 μg/mL increased the activity as a radical scavenger from 52.409% to 89.412%. According to Figure 6, the highest average antioxidant activity among sea urchin samples was associated with SSU, followed by CSU and GSU. All group differences were significant (*p* < 0.05).

## 4 Discussion

This study investigates the toxinological properties of different tissues of two Persian Gulf echinoderms, the brittle star *O. erinaceus* and the sea urchin *E. mathaei* extracts. In this regard, their acetyl and butyrylcholinesterase inhibitory activities, chemical composition and, antioxidant activity, were examined *in vitro* and *in silico*.

Few studies have examined the toxic effects of various sea urchin and brittle star extracts. Toxicity evaluations of the test chemicals are based on LD_50_ values. According to the classification by Loomis and Hayes (1996), substances with LD_50_ < 5 mg/kg are classified as “extremely toxic” and substances with an LD_50_ of 5–50, 50–500, 500–5,000 and 5,000–15,000 mg/kg are classified as " Highly toxic, moderately toxic, slightly toxic, and practically non-toxic, respectively, and 15,000 mg/kg and above are classified as relatively harmless (Loomis and Hayes, 1996). Based on this classification, the toxicities of all three sea urchin samples are extremely toxic and highly toxic for the brittle star. Furthermore, the observed disparities in LD_50_ values among distinct parts of the sea urchin strongly indicate the presence of varying toxic compounds within these anatomical regions. This inference aligns seamlessly with the findings from the GC-MS analysis, underscoring the potential significance of these variations in venom composition. Such insights hold considerable promise for shedding light on the ecological functions of these marine organisms and their prospective applications in pharmacological research.

In current study, GC-MS analysis of the BS, SSU, GSU and CSU samples showed 25, 12, 23, and 21 compounds including pyridines, piperidines, indole alkaloids, monoterpenes, xanthophylls, esters, sterols, alkanes, amides, carbohydrates, etc. These results in accordance with other studies focused on bioactivity of compounds derived from brittle stars and sea urchins (Nuzzo et al., 2017; Francis and Chakraborty, 2020a; Francis and Chakraborty, 2020b; Strobykina et al., 2020).

Molecular docking studies of these ligands with AChE and BChE enzymes (Table 5) showed that of the 25, 23, 21, and 12 compounds identified in BS, GSU, CSU, and SSU, respectively a number of 7, 13, 10, and 4 compounds with a binding energy between ligand and AChE, more than galantamine (−7.1 Kcal/mol), as well for BChE, a number of 4, 9, 8 and three compounds exhibited higher binding energies between the ligand and BChE than galantamine (−5.9 kcal/mol). Consequently, the *in vitro* results showing the highest AChE activity for GSU samples can be attributed to a greater number of compounds with strong interactions with AChE, especially **G**_**2**_. Furthermore, the highest inhibitory activity of **BS** against BChE *in vitro* can be attributed to the presence of 16 compounds with acceptable binding energy (>-4.0 kcal/mol), specifically **BS**_**4**_.

Compounds **BS**_**4**_, **S**_**5,**_ and **C**_**7**_ similarly showed the highest affinity for both enzymes. Furthermore, compounds **G**_**2**_ and **G**_**6**_ exhibited the highest binding energies for the AChE and BChE enzymes, respectively. Overall, **C**_**7**_ showed the highest inhibitory activity against both enzymes. Further discussion about these compounds follows.

**BS**_**4**_ and **G**_**2**_ are pyrazole found in brittle star and GSU samples, respectively. There are many reports on AChE and BChE activities of these compounds (Tarİkoğullari et al., 2016; Shchepochkin et al., 2023). Alkaloids are recognized as potential inhibitors of cholinesterase, offering promise for Alzheimer’s disease treatment (Konrath et al., 2013). **S**_**5**_ and **C**_**7**_ are indole alkaloids, the largest and most attractive tryptophan-derived alkaloids (Schardl et al., 2006). The potent *in vitro* anti-AChE activity of indole alkaloid harmine (**S**_**5**_), as a harmala alkaloid (IC_50_: 9.05 μM), was reported by He et al. (He et al., 2015) that is confirmed the results obtained in the present *in silico* study. **G**_**6**_, also referred to as citrinin, exhibits moderate inhibitory activity against AChE and BuChE, as determined by the Ellman’s method (with an IC_50_ value of 5.06 ± 0.15 μg/mL for AChE and 8.02 ± 0.08 μg/mL for BChE) (Hamed et al., 2023).

There are few reports on the antioxidant capacity of marine echinoderms (George et al., 2023; Klimenko et al., 2023). In this study, **BS** sample exhibited significantly higher antioxidant activity than the other samples. A similar study by Rahman et al. (2023) found that brittle star samples exhibited potent antioxidant activity. They attributed this to the abundance of antioxidant compounds such as polyhydroxylated sterols and phenolic compounds in samples from this marine organism (Rahman et al., 2021). A phytochemical study of *O. erinaceus* extract by Amini and Baharara, (2015) demonstrated the presence of saponin, phenolic and flavonoid compounds, as well as the dose-dependent antioxidant activity of brittle star extract (Amini and Baharara, 2015). Nami et al. (2019) evaluated the antioxidant activity of different solvent extracts including n-hexane, ethyl acetate, and methanol from the central disc and arms of the brittle star *O. erinaceus*. According to their results, the methanol extract showed the highest antioxidant activity (Nami et al., 2019). A study by Amini et al. (2015) investigated the antioxidant and invasive capacity of HeLa carcinoma cells exposed to brittle star crude saponins, which used their chemodefense against pathogens and predators. Their results showed that the extracted saponins possessed ABTS and DPPH scavenging properties with IC_50_ values of 604.5 and 1,012 μg/mL, respectively. Brittle star saponins markedly inhibited cervical cancer invasion and migration associated with the downregulation of matrix metalloproteinase expression. Therefore, saponins have been proposed as candidate antioxidants (Amini et al., 2015).

## 5 Conclusion

The potency of venoms is considered extremely toxic to all sea urchin specimens and highly toxic to brittle stars by LD_50_ classification. SDS-PAGE and total protein studies showed that at least part of the venom was proteinaceous. The highest total protein content was associated with coelomic fluid samples. GC-MS analysis of the identified samples revealed 12, 23, 21, and 25 compounds with different chemical and bioactive structures, including alkaloids, terpenes, and steroids, respectively for spine, gonad, and coelomic fluids of sea urchin and brittle star samples. Furthermore, the brittle star sample showed significantly higher antioxidant activity than other samples containing standard ascorbic acid at all concentrations tested. According to the results, both samples act as significant inhibitors of both AChE and BChE enzymes. In silico data for the identified compounds also supported the experimental results. The alkaloid compound **C_7_** showed the highest affinity for both enzymes. Further studies are needed to isolated this compound and determine whether compound **C**_**7**_ could be a therapeutic candidate for Alzheimer’s disease. Ultimately driving advancements in medical science is our primary goal as we work towards improving quality of life for individuals affected by such conditions.

## Data Availability

The datasets presented in this study can be found in online repositories. The names of the repository/repositories and accession number(s) can be found in the article/supplementary material.
